# Supplementary material for: Machine learning driven dashboard for chronic myeloid leukemia prediction using protein sequences
Source: PLoS One. 2025 Jun 18;20(6):e0321761. doi: 10.1371/journal.pone.0321761 (PMC12176232; doi:10.1371/journal.pone.0321761)
Supplement: Attachment — Submitted filename: Comments_PONE-D-24-31032.docx [file pone.0321761.s001.docx]

**Minor Comments:**

**Comment 1:** The Materials & Methods section at line 140 appears to be incomplete. Additionally, please include the details of the genes in the introduction. In the Materials & Methods section, simply mention the database used for the dataset collection, the keyword search conducted on UniProt, and the number of sequences obtained from UniProt.

**Comment 2:** I’m unclear on the need to explain the FASTA file format, as it is a widely known format commonly used in sequencing. Do not make another section for this just add in the dataset collection section.

**Comment 3:** In the "Sample of Protein Sequence (HSP90)" section, the total number of sequences before and after filtering is missing. Please include this information in the Materials & Methods section.

**Comment 4:** Please specify the training and validation datasets, including the number of sequences for each set and each protein. If possible, consider using a table to clearly present the total number of sequences used for training and validation for the BCL2, HSP90, PARP, and RB proteins.

**Comment 5:** It is recommended to perform 5-fold or 10-fold cross-validation on your internal dataset (training dataset) to enhance the reliability of your results.

**Comment 6:** In the results section, please include the performance metrics for the training and validation models for each protein. If the dataset is too small to make predictions for individual proteins, please explain the rationale behind merging different datasets.

**Comment 7:** It is recommended to separate the results and discussion sections. This would allow you to include other methods that perform similar analyses in the discussion section. Additionally, if you identify other methods that create similar dashboards, it would be valuable to include a comparison.

**Comment 8:** The quality of the images is very poor and needs to be improved.

**Comment 9:** The link to the dashboard app is missing. Additionally, please include a section in the Materials & Methods that outlines the architecture for creating this app.
